# Supplementary material for: EGFR-Targeted Antibody–Drug Conjugate to Different Aminobisphosphonates: Direct and Indirect Antitumor Effects on Colorectal Carcinoma Cells
Source: Cancers (Basel). 2024 Mar 22;16(7):1256. doi: 10.3390/cancers16071256 (PMC11011001; doi:10.3390/cancers16071256)
Supplement: Supplementary file 1 [file cancers-16-01256-s001.zip › cancers-2893706-supplementary.pdf]

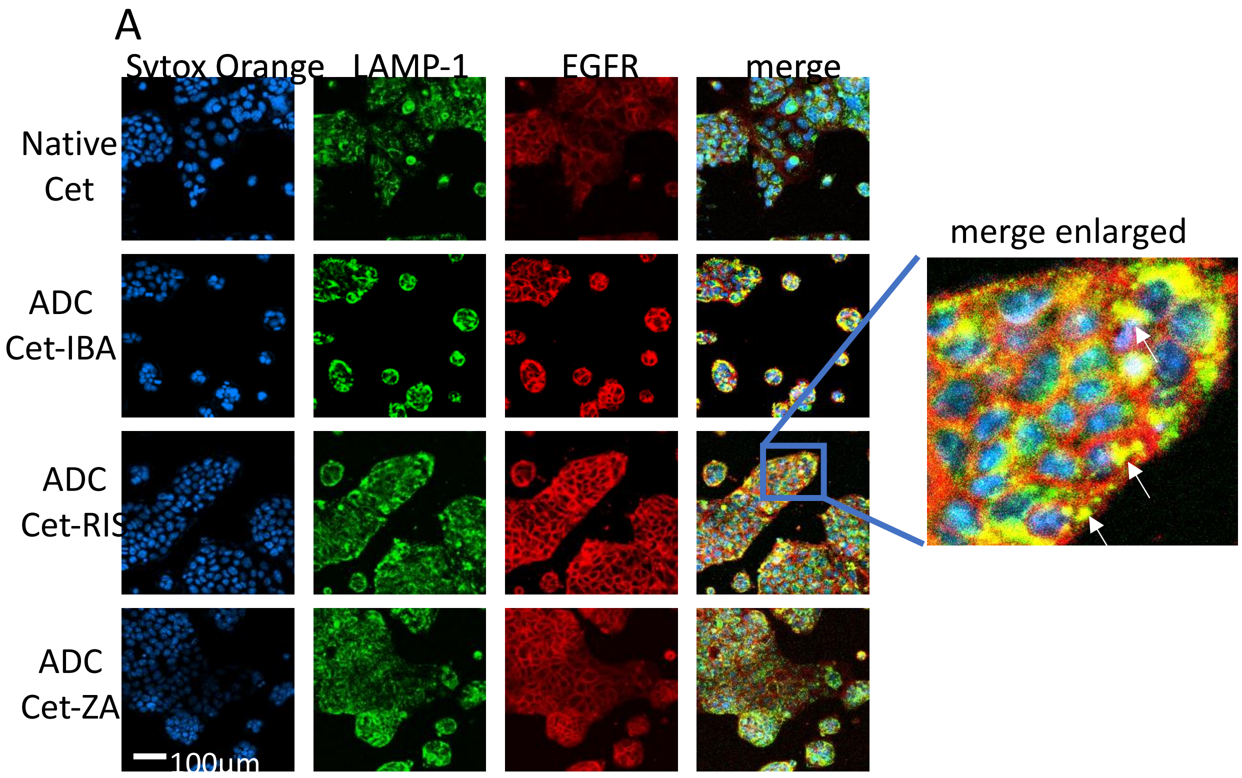
A
Sytox Orange
LAMP-1
EGFR
merge
Native
Cet
ADC
Cet-IBA
ADC
Cet-RIS
ADC
Cet-ZA
100µm
merge enlarged

Caco-2

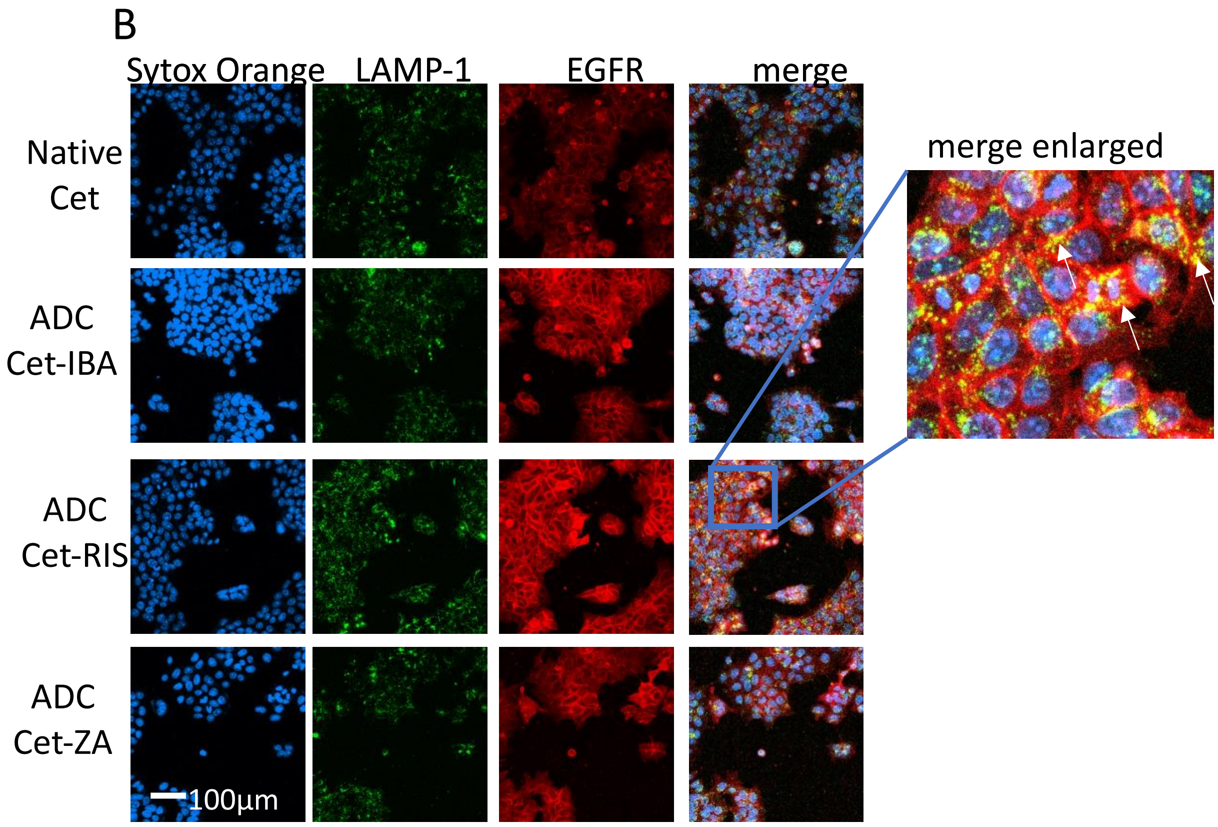
B
Sytox Orange
LAMP-1
EGFR
merge
Native
Cet
ADC
Cet-IBA
ADC
Cet-RIS
ADC
Cet-ZA
100µm
merge enlarged

DLD-1

Supplementary Figure S1. Internalization of EGFR in CRC cell lines. The Caco-2 (A) or the DLD-1 (B) cell line were seeded in image 96w flat bottomed plates for 24 h to allow adherence. Then, the cells were incubated with the native antibody Cet (upper images) or ADC (lower images, Cet-IBA or Cet-RIS or Cet-ZA) for additional 24h. After this period of time, cells were fixed and incubated with the anti-LAMP-1 antibody followed by the staining with alexfluor488 (green color, second line of images), alexafluor647 (red color, anti-human to detect EGFR antibody, third line of images) and Sytox Orange probe to identify nuclei (blue color, first line of images). Merge images represent the overlay of the three staining. Each image was taken at the confocal plane after scanning in sequenxce mode to avoid cross-talk among the different wavelength emission. The yellow region represents colocalization area for the indicated markers. Arrows in the merge enlarged show some yellows intracellular vesicles. Bar: 100µm, 200x magnification

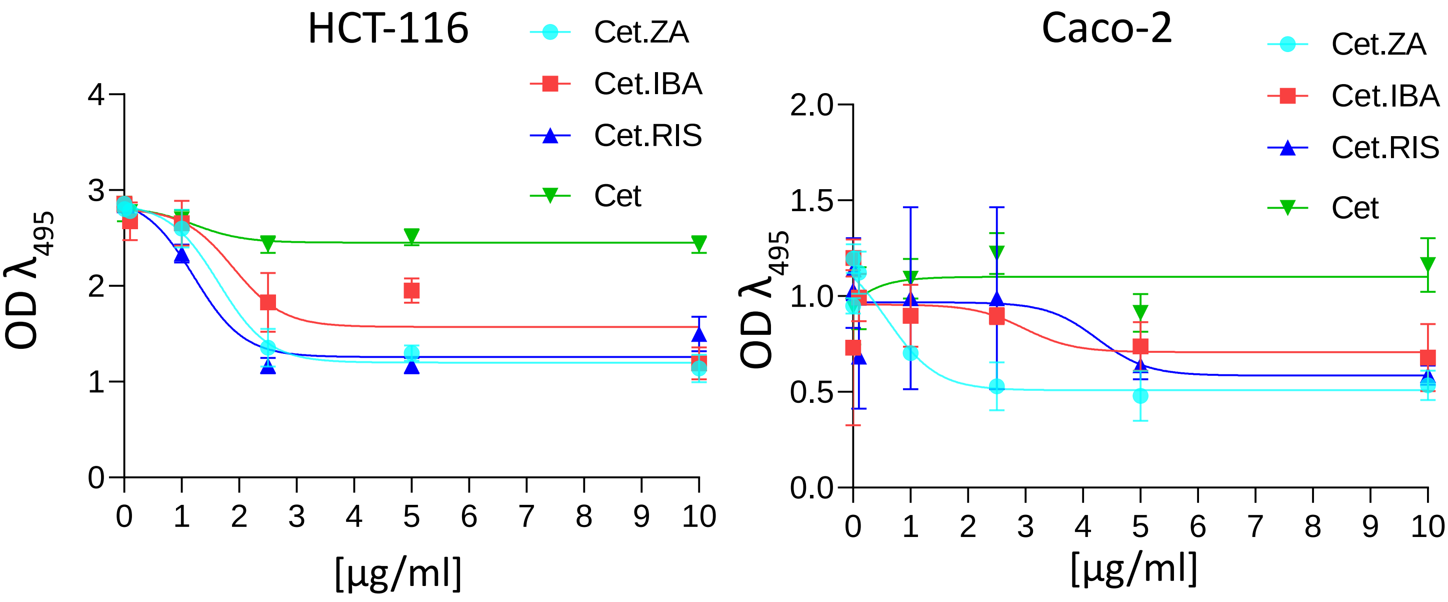

Supplementary Figure S2. IC_{50} of anti-EGFR antibody cetuximab linked to N-PBs. The indicated antibodies were used in a cytotoxic experiment using Vδ2T cells obtained from a healthy donor PBMC stimulated with 1µM of soluble ZA and IL-2 as described [30]. On day 21, Vδ2T cells (>95% of activated cells) were used at the effector: tumor target ratio of 3:1. Antibodies were used at different concentrations and the killing evaluated after the adherence of spheroids to conventional plates and staining with crystal violet. The decrease of OD in the presence of antibodies indicate the effective killing (i.e. reduced vitality).

Tumor cell lines were used as spheroids. The number of tumor cells in spheroids was calculated by ATP content of spheroids compared to the ATP content of a defined number of tumor cells in suspension.

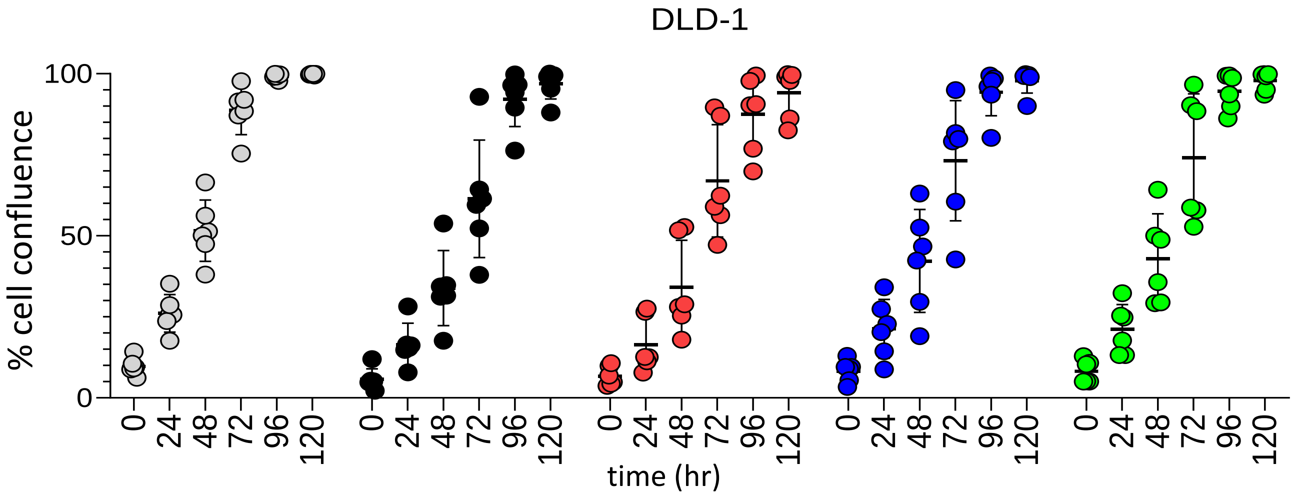

Supplementary Figure S3. Effect of ADC on DLD-1 CRC cell line proliferation. A. The DLD-1 cell line was seeded in flat-bottomed plates without (no antibody, gray circle) or with 2µg/ml of the indicated antibodies (Cet, black circle; Cet-IBA, red circle; Cet-RIS blue circle; Cet-ZA, green circle). Cell proliferation was assessed as percentage of confluency by taking images of culture wells at the indicated time points (0, 24, 48, 72, 96, 120hr). Each point correspond to six replicates for each experimental conditions. The percentage of confluency was calculated by the associated software (CellStudio) to Cell Cyte X plate scanner. The results are expressed as mean±SD of confluency of six replicate culture wells.

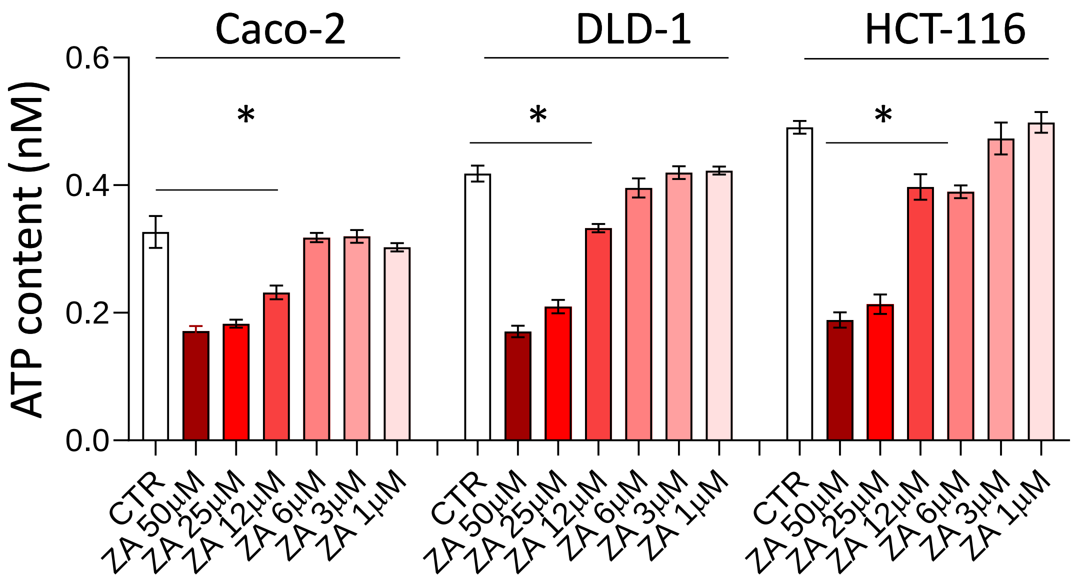

Supplementary Figure S4. Effect of soluble ZA on the cell proliferation of CRC cell lines. The indicated cell lines (Caco-2, DLD-1 and HCT-116) were seeded in conventional flat-bottomed culture plates at the starting number of $5x10^4$cells/well without (CTR) or with the indicated amount of ZA.

After 5 days of culture, the proliferation was assessed with Cell Titre Glow (Promega) evaluating the ATP content by luminescence. The results are expressed as nM of ATP determined performing titration with serial dilution of standard purified ATP and they are the mean of three replicates. * p<0.001 ZA 12μM vs CTR for Caco-2 and DLD-1 and ZA 6μM vs CTR.

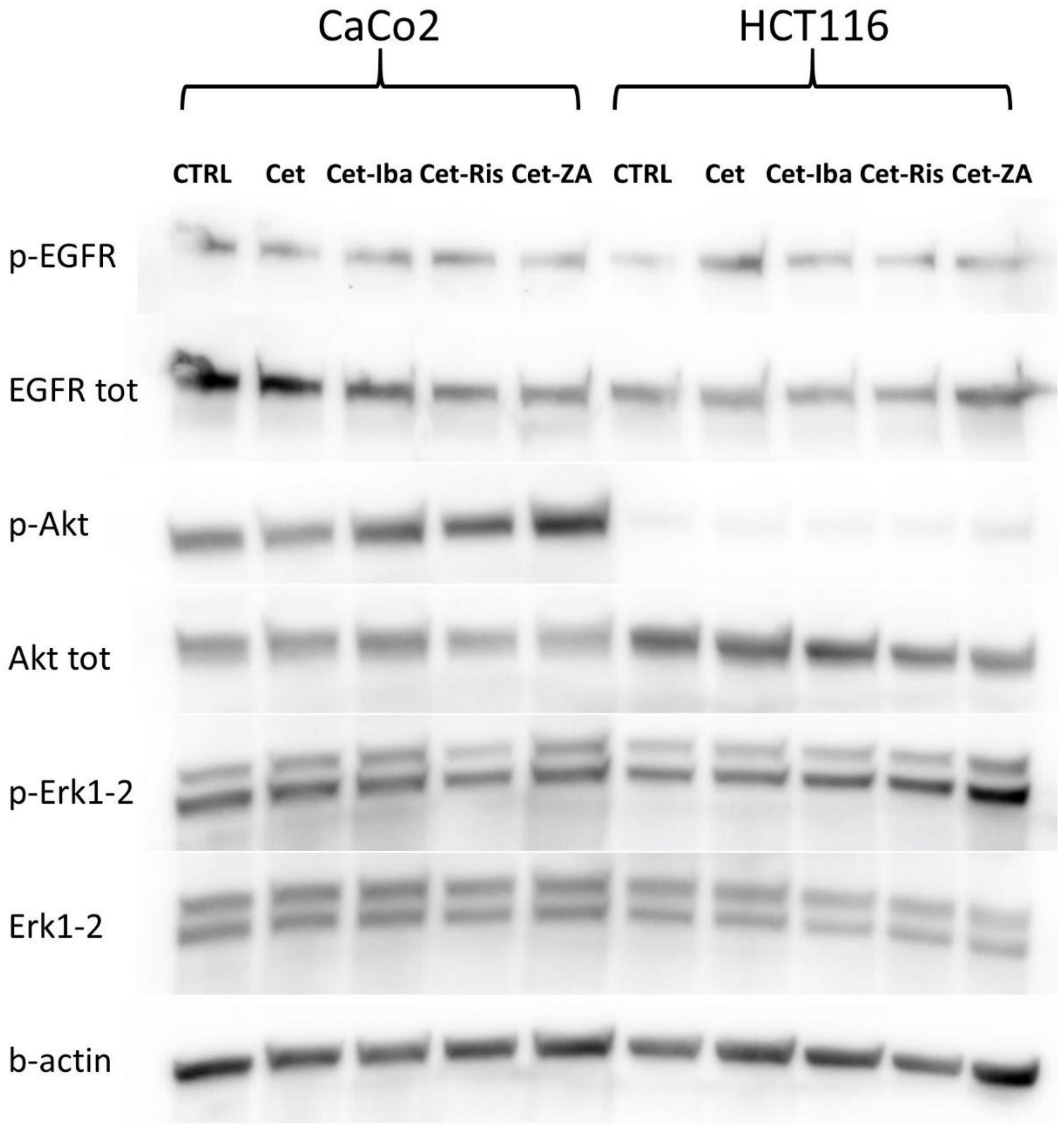

Supplementary Figure S5. EGFR and EGFR-associated downstream signaling molecules in CRC cell lines upon treatment with anti-EGFR antibodies. Caco-2 and HCT-116 cell lines cultured in complete medium were incubated without (CTR) or with 2μg/ml of the indicated antibodies for 24h. After this incubation period, cells were lysed and western blots were performed to analyse the amount of EGFR, Akt, Erk-1 and Erk-2 and the corresponding phosphorylated forms of these receptors and signalling molecules. β-actin was used as loading control.
